# Prognostic and predictive value of a mRNA signature in peripheral T‐cell lymphomas: A mRNA expression analysis

**DOI:** 10.1111/jcmm.15851

**Published:** 2020-12-01

**Authors:** Jiannan Tu, Zhixing Kuang, Xiaoliang Xie, Shizhen Wu, Ting Wu, Shengchi Chen

**Affiliations:** ^1^ Department of Oncology Nanping First Hospital Affiliated to Fujian Medical University Nanping China; ^2^ Department of Radiation Oncology Nanping First Hospital Affiliated to Fujian Medical University Nanping China; ^3^ Department of Orthopedics Shanghai Municipal Hospital of Traditional Chinese Medicine Shanghai University of Traditional Chinese Medicine Shanghai China

**Keywords:** mRNA, peripheral T‐cell lymphomas, predictive, Prognostic, signature

## Abstract

Current international prognostic index is widely questioned on the risk stratification of peripheral T‐cell lymphoma and does not accurately predict the outcome for patients. We postulated that multiple mRNAs could combine into a model to improve risk stratification and helping clinicians make treatment decisions. In this study, the gene expression profiles were downloaded from the Gene Expression Omnibus (GEO) database. Weighted gene co‐expression network analysis (WGCNA) was used to screening genes in selected module which most closely related to PTCLs, and then built a mRNA signature using a LASSO Cox regression model and validated the prognostic accuracy of it. Finally, a nomogram was constructed and the performance was assessed. A total of 799 WGCNA‐selected mRNAs in black module were identified, and a mRNA signature which based on DOCK2, GSTM1, H2AFY, KCNAB2, LAPTM5 and SYK for PTCLs was developed. Significantly statistical difference can be seen in overall survival of PTCLs between low‐risk group and high‐risk group (training set:hazard ratio [HR] 4.3, 95% CI 2.4‐7.4, *P* < .0001; internal testing set:hazard ratio [HR] 2.4, 95% CI 1.2‐4.8, *P* < .01; external testing set:hazard ratio [HR] 2.3, 95% CI 1.10‐4.7, *P* = .02). Furthermore, multivariate regression demonstrated that the signature was an independently prognostic factor. Moreover, the nomogram which combined the mRNA signature and multiple clinical factors suggesting that predicted survival probability agreed well with the actual survival probability. The signature is a reliable prognostic tool for patients with PTCLs, and it has the potential for clinicians to implement personalized therapeutic regimen for patients with PTCLs.

## 1 INTRODUCTION

Non‐Hodgkin lymphomas are clonal neoplasms that arise from lymphocyte at various stages of maturation,^1^ it estimated that 77 240 new cases of non‐Hodgkin lymphoma are expected in the United States, and 19 940 patents will die for this disease in 2020.^2^ Peripheral T‐cell lymphomas (PTCLs) are a subgroup of non‐Hodgkin lymphomas which also characterized as a infrequency and heterogeneous aggressive behaviour diseases that associated with very dismal prognosis, representing 10%–15% of non‐Hodgkin lymphomas (NHLs) in Western countries but up to 35% in some countries of Asian.^3^ Peripheral T‐cell lymphomas (PTCLs) comprise more than 30 distinct histologic subtypes including anaplastic lymphoma kinase (ALK)‐positive anaplastic large cell lymphoma (ALCL) and ALK‐negative ALCL, extranodal natural killer (NK)/T‐cell lymphoma (ENKTL), angioimmunoblastic T‐cell lymphoma(AITL), and PTCL, not otherwise specified (PTCL‐NOS) according to World Health Organization (WHO) classification system 2017.^4^ Numerous attempts have been made to optimize the treatment approach, but no definitive standard therapy has been reached.^5^ The traditionally combination regimens such as CHOP or a CHOP‐like regimen which initially established for aggressive B‐cell lymphomas are most widely used in PTCLs patient.^6^ However, outcomes for most patients treated with CHOP are still poor, with only 33%‐43% with PTCLs achieving a complete response (CR) and 5‐year overall survival (OS) barely exceeds achieving 38.5%.^7^ Given the poor outcomes in PTCLs, several novel drugs such as pralatrexate, Mogamulizumab, Chidamide, romidepsin, brentuximab vedotin, and Forodesine have been approved by FDA for the treatment of relapsed and refractory PTCLs recently,^8^ but none of these new drugs led to improvement of survival.^9^, ^10^ Moreover, the role of stem‐cell transplantation for PTCLs remains controversial in front‐line settings.^11^ There may be a role for prognostic biomarkers in risk classification of PTCLs patients. High‐risk patients could receive more intensive treatment to avoid insufficient treatment, whereas low‐risk patients should choose low‐intensity treatment regime to avoid excessive drug toxicity. Therefore, it is urgent to identify robust biomarkers for predict the prognosis of PTCLs, and discriminate patients who might benefit from the therapy.

To date, the most widely used model for evaluating the prognosis of peripheral T‐cell lymphoma is international prognostic index (IPI) that based on performance status, lactate dehydrogenase, extranodal involvement, stage and age, which was initially established for diffuse large B‐cell lymphoma (DLBCL). However, Given the marked heterogeneity among the patients that diagnosed with PTCLs, the IPI score is far less satisfactory for distinguishing recurrence risk for PTCLs patients than for aggressive B‐cell lymphoma.^12^ For example, even patients which categorized in the best risk group (IPI 0) still experience an extremely unfavourable outcome, the cause of this phenomenon is attributed to that IPI score only focused on clinical characteristics, with very few genomic information reflecting the molecular mechanism underlying the PTCLs biology. On the other hand, the lack of information on risk stratification brings the merits of limitations for clinicians to conduct individualized treatment strategies. Recently, several gene expression biomarker signatures that based on gene expression profiling (GEP) and whole‐genome methylation profiling have been build and used to predict the prognosis of human cancer,^13^, ^14^, ^15^, ^16^ but none mRNA signatures have been utilized for PTCLs patients.

Weighted gene co‐expression network analysis (WGCNA) is powerful screening approach and has been gradually valued in discovery of novel biomarkers or therapeutic targets via construct free‐scale gene co‐expression networks.^17^ In this study, we explore the correlation between PTCLs and gene sets by WGCNA. Furthermore, the univariate proportional hazards analysis and LASSO Cox regression were carried out to identify a mRNA signature which beyond clinical parameters and significant associated with PTCLs prognosis. Finally, a prognostic nomogram was established based on the combination of signature and clinical characteristics.

## 2 MATERIALS AND METHODS

### 2.1 Data sources and data processing

**Figure 1:**
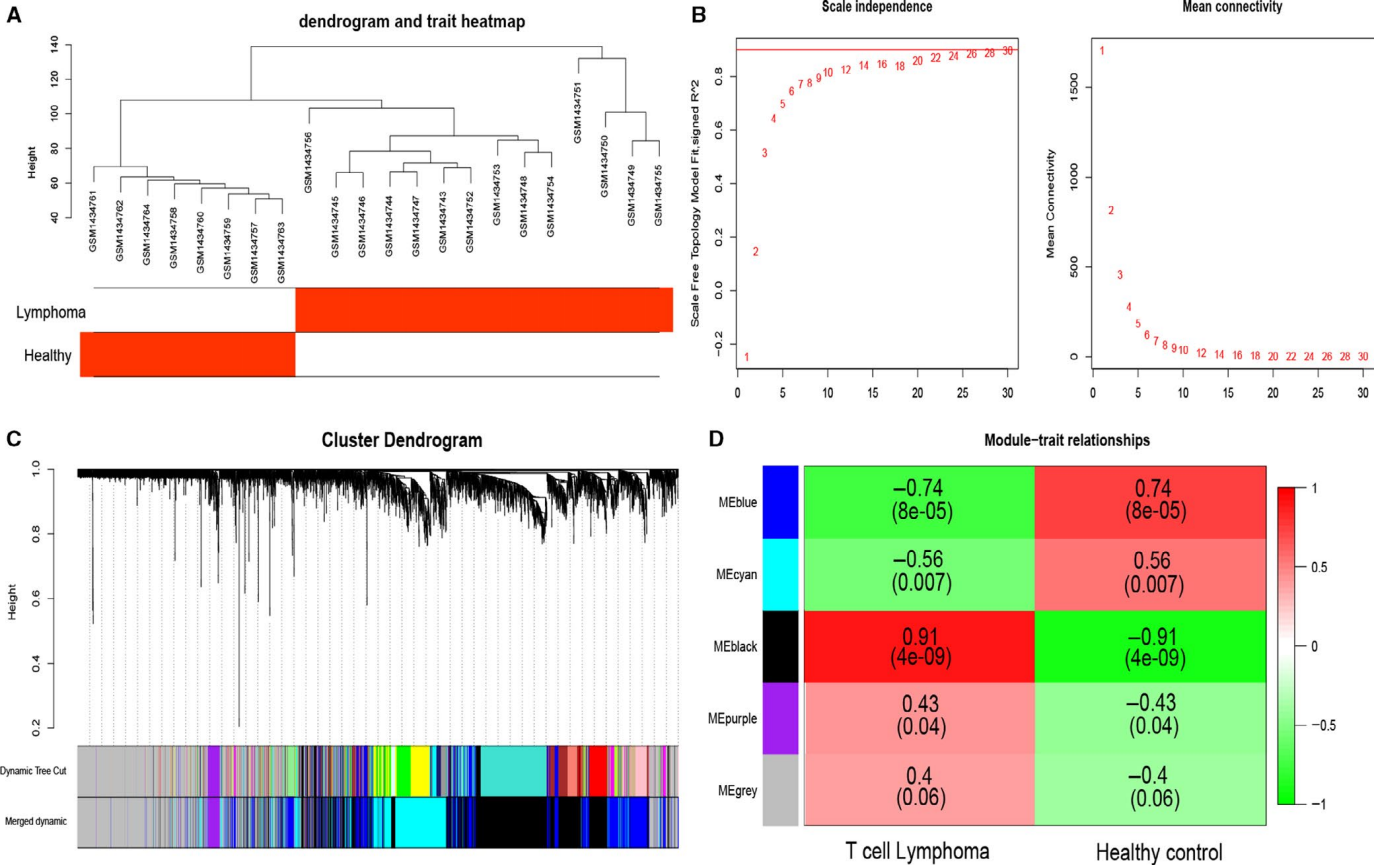
Identification of candidate genes in PTCLs. A, Clustering dendrogram of PTCLs and normal control. B, analysis of scale‐free fit for soft‐thresholding powers and 28 were selected as the best value. C, Dendrogram of genes clustered on a dissimilarity measure. D, Heat map of the relationships between modules and PTCLs by Pearson correlation

The raw data of GSE59307, GSE58445, GSE19069, GSE90597 and GSE53798 were downloaded from the Gene Expression Omnibus (GEO; http://www.ncbi.nlm.nih.gov/geo/) database, and all datasets except GSE90597 were built based on the GPL570 platform [HG‐U133_Plus_2]. A total of 14 samples of cutaneous T‐cell lymphoma (CTCL) and 8 cases of healthy control specimens were obtained in GSE59307 (**Figure** **1A** **)**, whereas the GSE58445 and GSE19069 comprises 193 and 137 samples of PTCLs, respectively. In addition to this, 66 cases of ENKTL which is a subtype of PTCLs were included in GSE90597 and GSE53798 comprise by 26 cases of Diffuse large B‐cell lymphoma.According to current WHO classification, CTCL is a subtype of PTCLs; therefore, GSE59307 was chosen to construct the co‐expression network. The packages of 'simpleaffy',^18^ 'affyPLM' and 'arrayQualityMetrics' were utilized to perform the process of quality assessment (QA), quality control (QC), background correction and normalization. The probe id in datasets which based on GPL570 platform was annotated by the 'hgu133Plus2' package, and probe id of GSE90597 was annotated by GPL10739 files.

### 2.2 Co‐expression network construction

The top 5000 variant of expression profiles in GSE59307 were used to construct a co‐expression network by using the package of WGCNA, and the network topology was analysed with soft‐threshold power from 1 to 30. After determining the optimal beta value for the soft threshold parameter, the relational matrix can be converted into adjacent matrix, and then, it can be transformed into topological overlap matrix (TOM). Finally, average linkage hierarchical clustering was conducted to classify the highly correlated into modules according to the measure of TOM‐based dissimilarity measure.

### 2.3 Clinically significant modules visualization and identity the hub genes

To identify the modules which are significantly related to PTCLs, the module eigengene (ME) was used to characterize the expression profiles of each module and the correlation between PTCLs. The relationship of each genes with PTCLs was measured by gene significance (GS) value. Module significance (MS) represents the average GS of all genes that in the module. Finally, the module that highly related to PTCLs was chosen for further analysis. The connectivity of genes in module was quantified by the absolute value of the Pearson's correlation, and the module membership (MM) was defined as the correlation of module eigengenes (MEs) with genes. In present study, the intramodular hub genes were chosen with the criterion of GS > 0.2 and MM > 0.8 to ensure the reliability of the results.

### 2.4 LASSO Cox Regression conduction and identification of a mRNA signature

Univariate Cox proportional hazards regression analysis was applied to assess the relationship between the expression of WGCNA‐selected genes and the overall survival (OS) of patients with PTCLs, genes which calculated with *P* < .05 were sorted out and chosen to screening the most valuable predictable mRNAs by performing the LASSO Cox regression analysis which depends on the R package 'glmnet'.The optimal values of the penalty parameter λ were estimated through 10‐time cross‐validations. The risk score of mRNA signature for each patient was calculated by the coefficient that from LASSO regression analysis and expression level of each mRNA. The risk score was constructed as follows:

$$\text{Risk score}=\sum_{a=1}^{n}\exp_{a}*\beta_{a}$$

*n* was the number of prognostic genes, exp_a_ was the expression value of gene a, and *β* was the regression coefficient. All PTCLs patients were separated into high‐ and low‐risk groups according to median risk score that used as cut‐off value. Kaplan‐Meier estimator was carried out to assess the prognostic value of the mRNA signature. Survival prediction based on the risk score was illustrated by using the 'survivalROC' package. Wilcoxon signed‐rank was applied to compare the differential expression between high‐risk group and low‐risk group of PTCLs. In addition, the protein expression levels of the six genes in the mRNA signature were validated by immunohistochemistry through using the Human Protein Atlas database (https://www.proteinatlas.org/) and their mutation status was explored by cbioportal database (https://www.cbioportal.org/).

### 2.5 Immune landscape difference between high‐risk and low‐risk PTCLs patients

CIBERSORT is a tool based on the principle of linear support vector regression to deconvolute the expression matrix and calculate the proportions of 22 types of infiltrating immune cells in each sample.^19^ And only if P value of each sample less than 0.05 will be retained for subsequent analysis. Considering the small sample size of GSE90597, we combined the two datasets of GSE19069 and GSE90597 and used the combat function of the R software package of sva to remove batch effects and calculate the distribution of immune cells.^20^ Then, we analysed the immune differences between the high‐risk and low‐risk groups in the combined dataset and the GSE58445 dataset.

### 2.6 Integrated analysis by combining the clinical factors and mRNA signature

To investigate the effect of the risk signature on the prognosis of PTCLs patients, univariate and multivariate Cox regression analyses were conducted. The risk scores of six‐mRNA signature and other clinical characteristics, including gender and age, were used as covariates. Moreover, the six mRNA which screened by LASSO Cox regression also were selected as candidate mRNAs to explore the difference in survival between high and low expression groups which performed by Kaplan‐Meier survival analysis. Furthermore, the analysis concerning the correlation between risk score and currently available clinical characteristics was conducted.

### 2.7 Association of a mRNA signature and response to chemotherapy

The dataset of GSE53798 was originally established for predicting sensitivity to chemotherapy drugs in CHOP (cyclophosphamide, doxorubicin hydrochloride, vincristine, prednisone) regime for diffuse large B‐cell lymphoma cell.^21^ Considering that the degree of malignancy of diffuse large B‐cell lymphoma is comparable to that of some subtype of peripheral T‐cell lymphoma, and currently treatment of peripheral T‐cell lymphoma is similarly to which used for diffuse large B‐cell lymphoma (DLBCL). So, in this part, we investigated whether the mRNA signature could predict patients' responses to chemotherapy of vincristine.

### 2.8 Nomogram development and validation

The Cox regression model was used to perform the multivariable survival analysis and build nomograms. Calibration curves were selected to assess the consistency between the actual survival and the predicted survival for the nomogram. Nomogram and calibration curves were performed with the package named rms. The C‐index was utilized to measure the discrimination of the nomogram.

## 3 RESULTS

### 3.1 Pre‐processing of the datasets

All microarray data were converted into expression matrix after processing, 31 cases in GSE58445 and 11 cases in GSE90597 which lacking survival data were excluded in this study. In addition, after excluding unqualified samples, 162 patients in GSE58445 were randomly divided into the training set (*n* = 98) and testing set (*n* = 64) according to a ratio of 6:4.

### 3.2 Construction of weighted co‐expression network and identification of key modules

To ensure build a scale‐free network, the power of *β* = 28 (scale‐free *R*2 = 0.84) was selected as the best soft‐thresholding parameter (Figure 1B). Next, co‐expression modules were produced by method of dynamic tree cutting and make sure that the number of genes in each module is not <30 (Figure 1C). Additionally, by setting the parameter of MEDissThresas as 0.25, the modules that closely associated were merged into a larger one. Ultimately, there are five modules were generated in co‐expression network, and black module demonstrated the strongest positive correlation with PTCLs samples (weighted correlation = 0.91, *P* = 4e −9) (Figure 1D).

### 3.3 Identification of the six‐mRNA signature in training group patients

**Figure 2:**
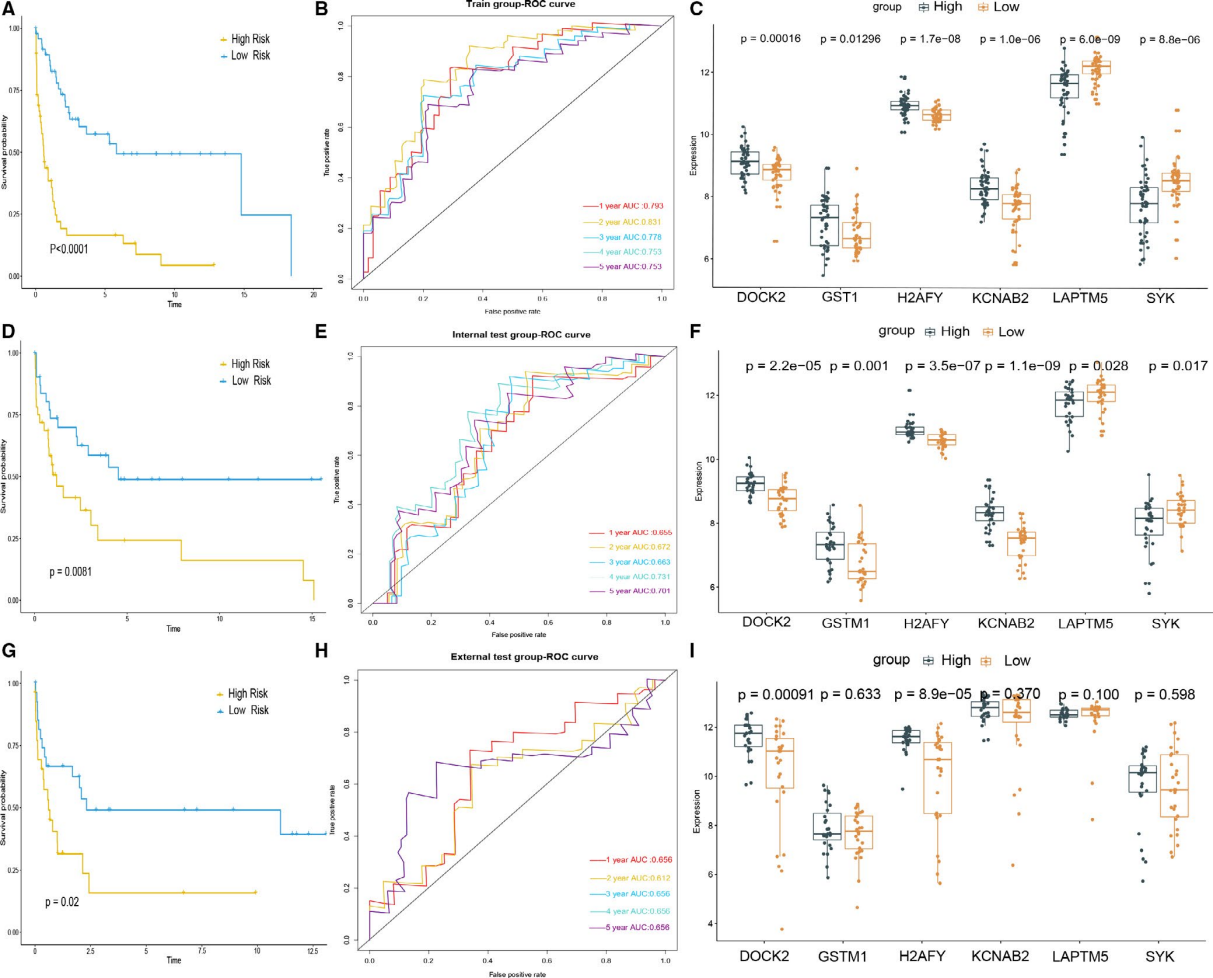
Prognostic and predictive value of the mRNA signature. A,D,G, Kaplan‐Meier survival curves for training group, internal testing group and external testing cohort of PTCLs patients. B,E,H, Time‐dependent ROC curves of 1, 2, 3, 4, 5 years for the six‐mRNA signature in training group, testing group and all cohort. C,F,I, Box plot visualization of the expression levels of DOCK2, GSTM1, H2AFY, KCNAB2, LAPTM5 and SYK in different risk group

All 799 WGCNA‐selected hub genes used to identify survival‐related mRNA by univariable Cox survival analysis in training group dataset, 15 genes were pre‐filtered based on *P* values < .05, and then, those genes were selected to preform LASSO Cox regression analysis in GSE58445 cohort (Figure S1). The risk score for predicting the outcome of patients was calculated with the following formula which based on the six mRNA: risk score = (0.2554 × DOCK2 expression) + (0.2334 × GSTM1 expression) + (0.3123 × H2AFY expression) + (0.1719 × KCNAB2 expression) + (−0.2820 × LAPTM5 expression) + (−0.1399 × SYK expression). According to the median of the risk score, all PTCLs patients were divided into high‐risk (n = 49) and low‐risk groups (n = 49). 5‐year os was 12.2% for the high‐risk group and 32.6% for the low‐risk group, which were significantly different in terms of overall survival(OS) ([HR] :5.6, 95% CI 2.75‐11.6, *P* < .0001).The 1‐year, 2‐year, 3‐year, 4‐year and 5‐year areas under the curve were 0.793, 0.831, 0.778, 0.753 and 0.753, respectively (Figure 2B). Additionally, the mRNA signature can function as a novel indicator of the survival of PTCLs patients, which was confirmed by Kaplan‐Meier curves (Figure 2A). Among these six mRNA, DOCK2, GSTM1, H2AFY and KCNAB2 significantly overexpressed in high‐risk PTCLs patients compare to high‐risk group and were associated with poor prognosis; LAPTM5 and SYK significantly overexpressed in low‐risk patients compare to high‐risk patients and related to prolonged prognosis(Figure 2C).

### 3.4 Validation of prognostic and predictive accuracy of the six‐mRNA signature in the internal and external testing group

The prognostic value of six‐mRNA signature was further evaluated in the internal test set and external testing set. In the internal testing cohort, the PTCLs were categorized 32 (50%) of 64 patients into the low‐risk group and 32 patients (50%) into the high‐risk group, and 5‐year os was 9.37% for the high‐risk group and 25% for the low‐risk group, which were significantly different in terms of overall survival(OS) ([HR] :2.4, 95% CI 1.2‐4.8, *P* < .01. Figure 2D). In the external testing cohort, the ENKTL which is a subtype of PTCLs was categorized 27 (49.09%) of 55 patients into the low‐risk group and 28 patients (50.91%) into the high‐risk group, and 5‐year os was 7.4% for the high‐risk group and 28.57% for the low‐risk group, ([HR] 2.3, 95% CI 1.10‐4.7, *P* = .02. Figure 2G). We also noted similar results in the total set of GSE58445, and 5‐year os was 11.1% for the high‐risk group and 29.6% for the low‐risk group ([HR]: 3.3, 95% CI 2.2‐5.0, *P* < .0001 Figure S2). Prognostic accuracy of the six‐mRNA based signature is also assessed by time‐dependent ROC analysis. The 1‐year, 2‐year, 3‐year, 4‐year and 5‐year areas under the curve for internal testing group were 0.655, 0.672, 0.663, 0.731 and 0.701 Figure 2E). Similarly, the 1‐year, 2‐year, 3‐year, 4‐year and 5‐year areas under the curve for external testing cohort set were 0.656, 0.612, 0.656, 0.656 and 0.656, respectively (Figure 2H).

**Figure 3:**
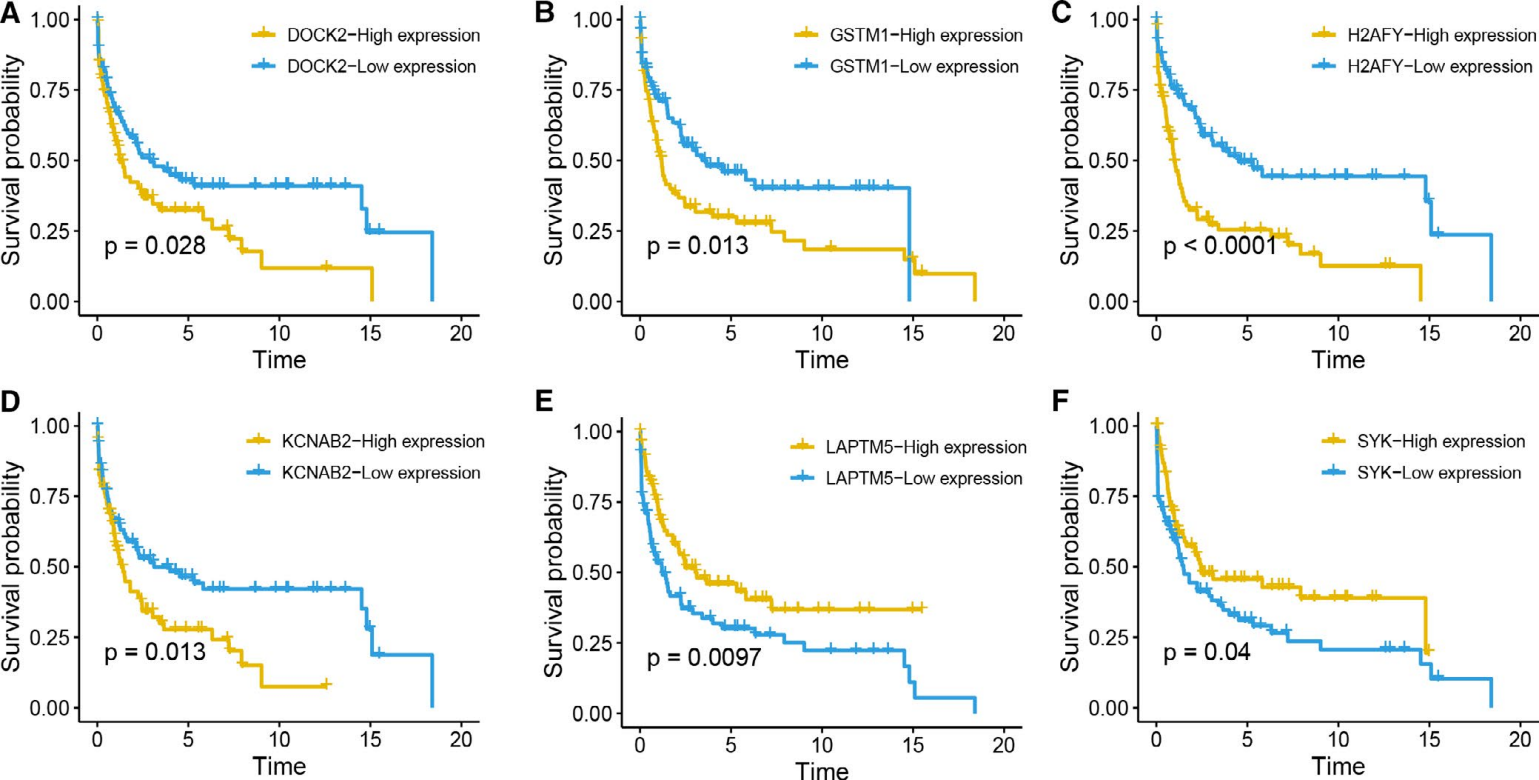
Survival analysis of DOCK2, GSTM1, H2AFY, KCNAB2, LAPTM5 and SYK in GSE58445 PTCLs cohorts (A, DOCK2; B, GSTM1; C, H2AFY; D, KCNAB2; E, LAPTM5; F, SYK)

In the internal verification cohort, the expression distribution of 6 genes between the high and low two groups is consistent with the test group (Figure 2F). In the external verification cohort, DOCK2 and H2AFY significantly overexpressed in high‐risk PTCLs patients compare to high‐risk group and there is no statisticaldifference in the expression level of the remaining four genes between the high and low two groups (Figure 2I). Moreover, we explored the impact of expression of these six mRNA on the prognosis of all PTCLs, and the median expression value of the selected genes was set up as cut‐off value in GSE58445, and fond high expression of LAPTM5 and SYK is a protective factor for prognosis of PTCLs; however, high expression of DOCK2, GSTM1, H2AFY and KCNAB2 is a risk factor to prognosis (Figure 3). Furthermore, we analysed the correlation between the expression of six genes and survival in GSE90597, and X‐tile software was used to find the best cut‐off value. We found that the high expression of GSTM1, H2AFY and KCNAB2 is negatively correlated with the prognosis, and LAPTM5 and SYK are positively correlated with the prognosis, which in line with the result in dataset of GSE58445 (Figure S3).

### 3.5 External validation of the protein expression levels and genetic alteration of the six mRNA

**Figure 4:**
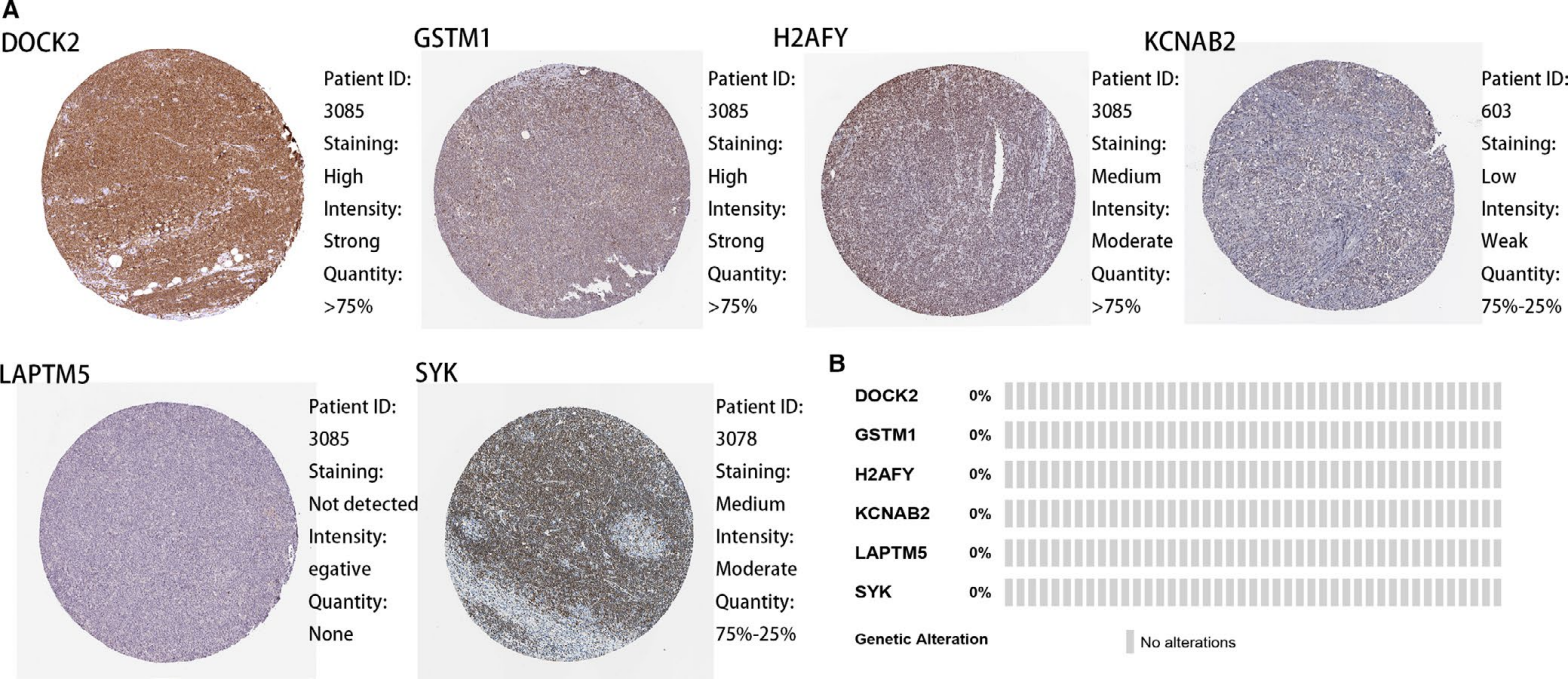
Expression and genetic alterations of the four predictive genes. A, The representative protein expression of the six mRNA in PTCLs. Data were from the Human Protein Atlas (http://www.proteinatlas.org) online database. B, Genetic alterations of the six mRNA in PTCLs

Protein expression levels in PTCLs which obtained and visualized by Human Protein Atlas database showed that DOCK, GSTM1, H2AFY and SYK represent medium to high degree positive in immunohistochemistry staining results. However, KCNAB2 shows weak positive and LAPTM5 shows negative staining (Figure 4A). All the results of protein expression levels are basically consistent with genes coefficient in our mRNA results. Among the 43 PTCLs patients enrolled in Cancer Genomics database of cBioportal, all six mRNA have no genetic alterations (Figure 4B).

### 3.6 Distribution of immune cells in different risk groups

**Figure 5:**
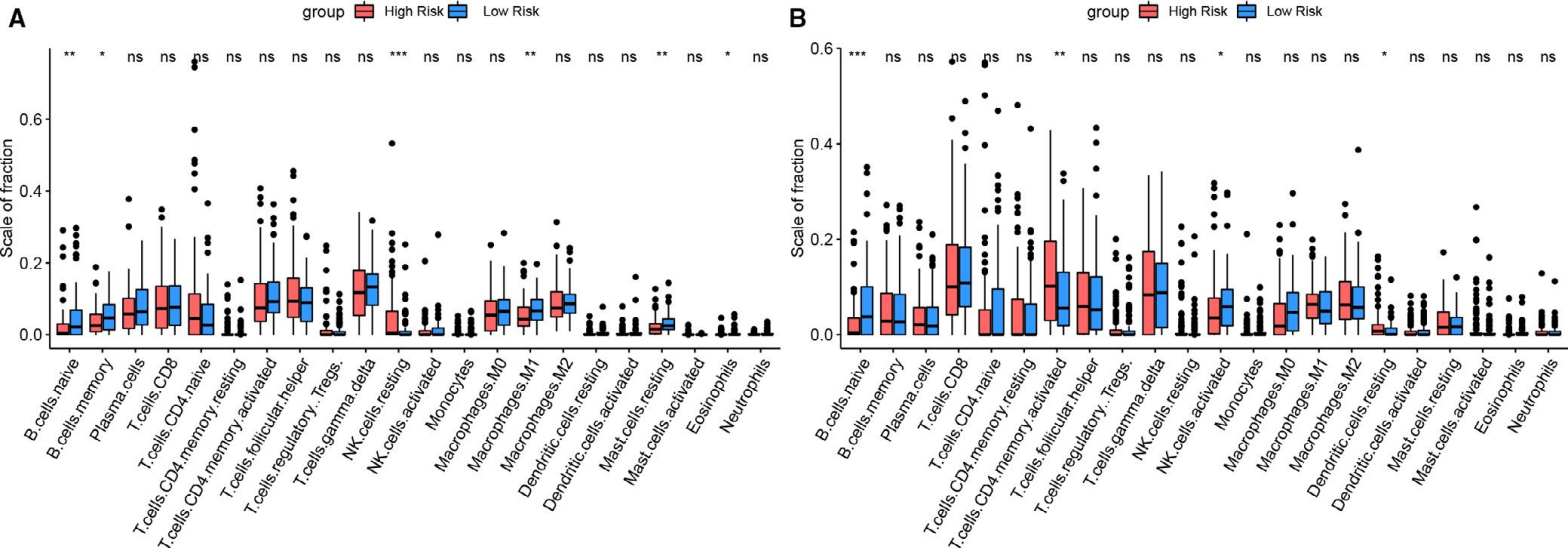
Differential distribution of immune cells between the high risk and low risk group. A, GSE58445 dataset. B, Combinational cohort which incorporated by GSE19069 and GSE90597 dataset

After completion of CIBERSORT immune analysis, we found that the two cohorts of PTCLs patients generally have similar immune cell distribution; additionally, naive B cells are statistically different in the high‐risk and low‐risk groups in both two cohorts. In the GSE58445 cohort, 81 cases PTCLs in each of the low‐risk group and the high‐risk group showed a significant difference in the presence of 6 immune cells types (naive B cells, memory B cells, resting natural killer cells, M1 macrophages, resting mast cells, eosinophils) (Figure 5A).In the combinational cohort which incorporated by GSE19069 and GSE90597 dataset, 99 cases PTCLs in the low‐risk group and 92 PTCLs cases in the high‐risk group showed a significant difference in distribution of 4 immune cells types (naive B cells, activated CD4 + T cells, activated NK cells and resting dendritic cells) (Figure 5B).

### 3.7 Independent prognostic role of the mRNA signature

To confirm the value of mRNA signature in assessing PTCLs patients’ prognosis, we performed univariate and multivariate Cox regression analyses in training group and testing dataset by including age, gender and mRNA signature as explanatory variables. Clinical characteristic parameters were grouped according to the International Prognostic Score (IPI) criteria: Age ≥ 60 Years. In training group, gender and mRNA signature were significantly correlated with OS by using univariate Cox regression. After multivariate adjustment using the factors above, the mRNA signature remained a powerful and independent prognostic factor for PTCLs patients (mRNA signature: HR = 5.6, 95% CI = 2.75‐11.6, *P* < .0001) (Table 1), the same results were also seen in testing group (internal testing cohort: HR = 2.7, 95% CI = 1.31‐5.6, *P *= .0007; external testing cohort: HR = 2.64, 95% CI = 2.64, *P* = .02),suggesting that the risk prognostic signature independent impact on prognostic of PTCLs patients.

**Table 1:** Univariate and multivariate Cox regression analyses of the mRNA signature in PTCLs patients

Variable	Univariate analysis			Multivariate analysis		
	HR	95% CI P	*P*	HR	95% CI	*P*
Training group						
Risk (High/Low)	4.30	2.40‐7.40	<.001	5.60	2.75‐11.6	<.001
Gender (Male/Female)	3.00	1.50‐5.90	.0016	1.90	0.88‐3.90	.10
Age (≥60/<60 y)	1.20	0.61‐2.10	0.65	1.70	0.88‐3.30	.118
Internal testing group						
Risk (High/Low)	2.40	1.20‐4.80	.01	2.70	1.31‐5.60	.007
Gender (Male/Female)	0.69	0.33‐1.40	.32	0.82	0.39‐1.80	.62
Age (≥60/<60 y)	1.70	0.82‐3.60	.15	1.86	0.89‐3.9	.10
External testing group						
Risk (High/Low)	2.30	1.10‐4.70	.023	2.64	1.16‐62	.02
Gender (Male/Female)	1.70	0.75‐3.70	.21	1.67	0.70‐3.95	.24
Age (≥60/<60 y)	3.00	1.40‐6.10	.003	3.15	1.41‐7.02	.005

### 3.8 Association of the mRNA risk score with Clinical characteristics and the role of risk stratification on response to chemotherapy

**Figure 6:**
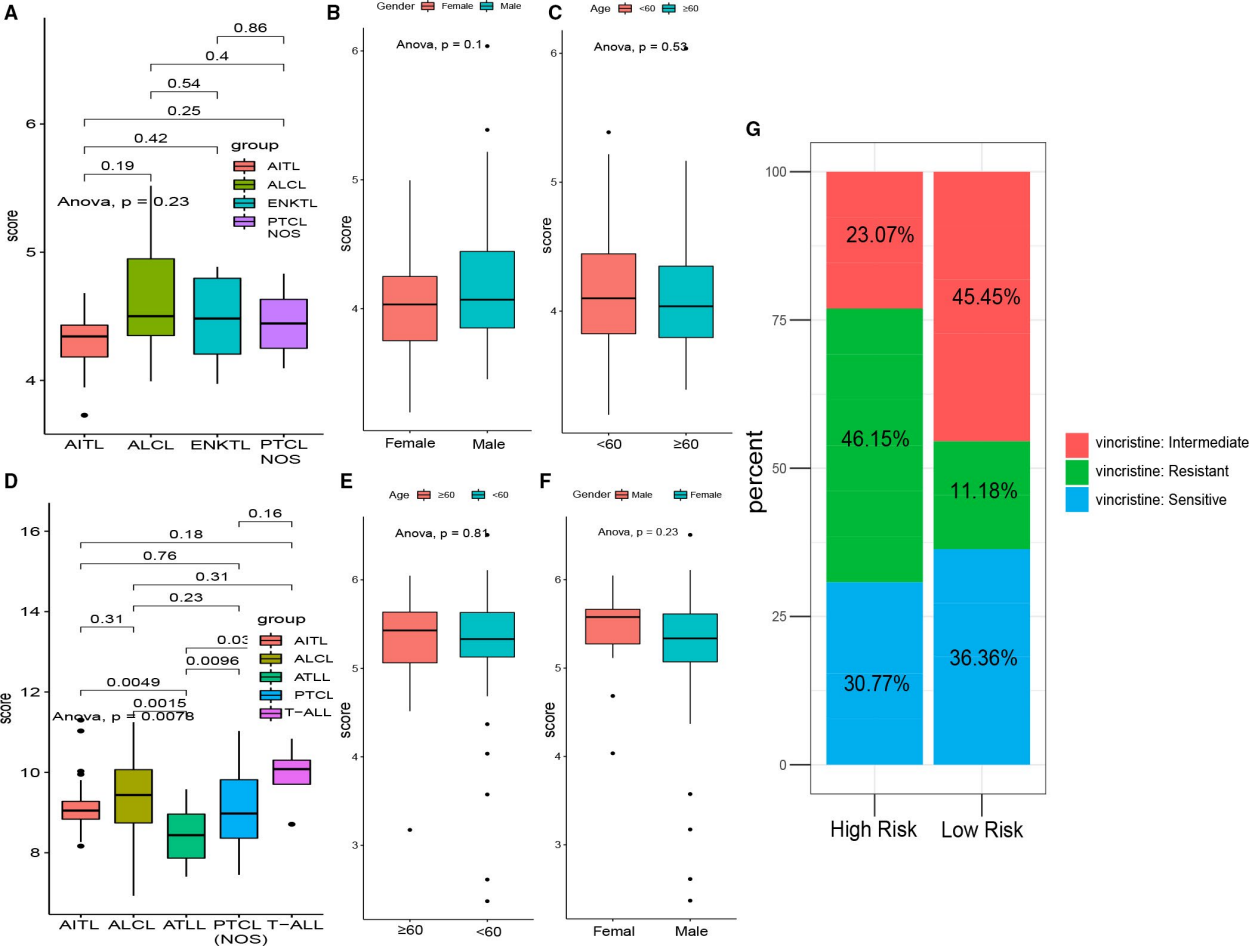
Distribution of the mRNA risk score in distinct clinical characteristics and the role of risk stratification on response to chemotherapy. A,D, Differences in risk score among different PTCLs subtypes. B,E, The risk score was group by age. C,F, The risk score was group by gender. G, Risk stratification on response to vincristine chemotherapy

In order to figure out the impact of different PTCLs subtypes and clinical features on risk scores, we analysed the risk differences between samples with different pathological types and different clinical characteristics according to the available information in the GEO dataset. The risk score of AITL, ALTL, ENKTL and PTCL‐NOS in dataset of GSE58445 has no obvious difference (Figure 6A), as the same results of status of gender and age impact on risk score (Figure 6B,C). Compared with other types of lymphoma, adult T‐cell leukaemia/lymphoma (ATLL) has the lowest risk score and T‐cell leukaemia/lymphoma (T‐ALL) has the highest score in dataset of GSE19069(Figure 6D), and there is no statistical difference in risk score between different ages and different genders in GSE19069(Figure 6E,F). Apart from this, we investigated whether the mRNA signature could predict patients’ response to chemotherapy in GSE53798. More patients with high risk exhibited resistant to vincristine compare to low‐risk patients (Figure 6G), which may partly explain why high‐risk PTCLs patients have a worse prognosis than low‐risk PTCLs patients.

### 3.9 Establishment of the nomogram and assessment of predictive value of mRNA signature

**Figure 7:**
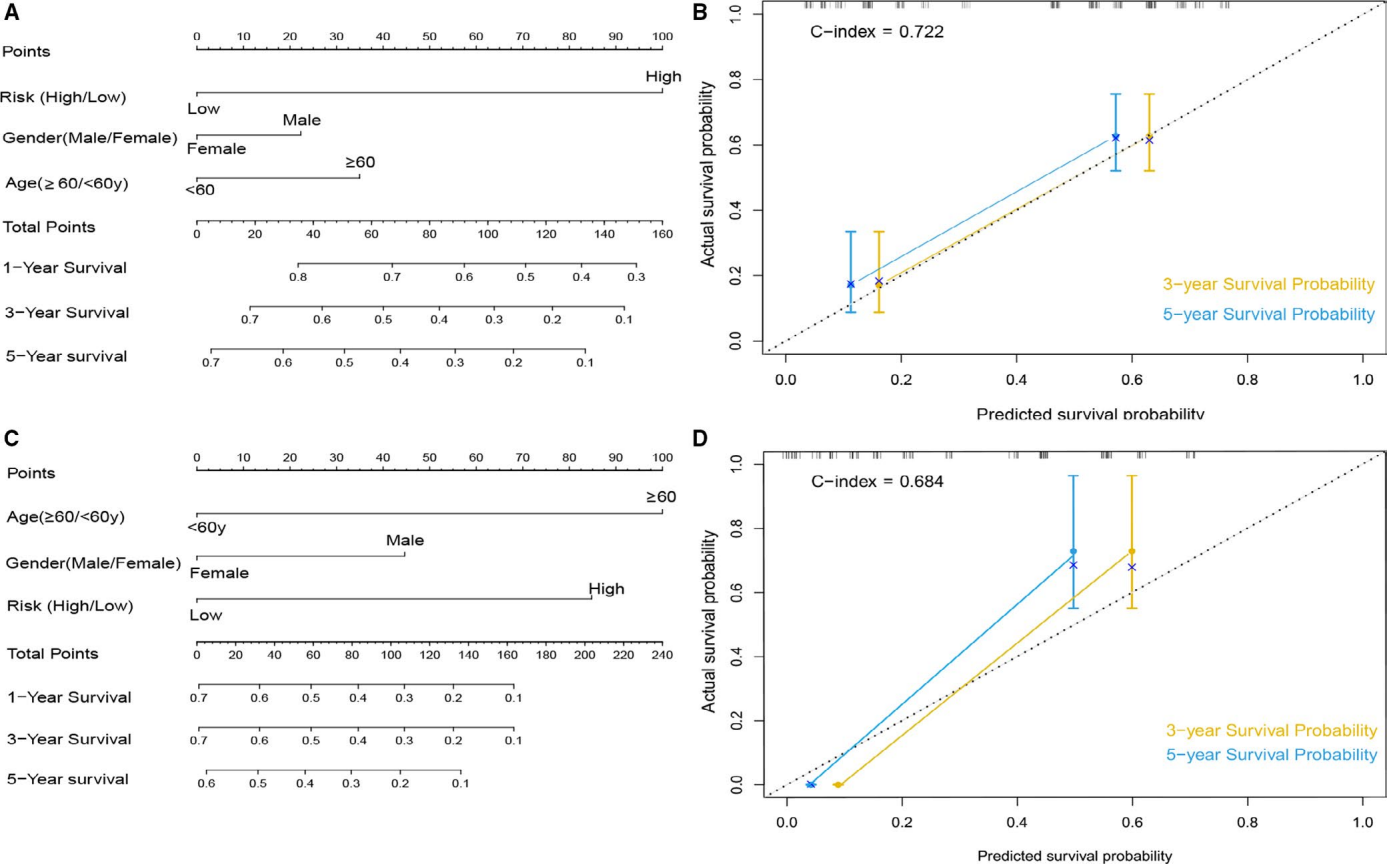
Nomogram and calibration plot for GSE58445 cohort and GSE90597 cohort. (A,C) The nomogram was constructed for predicting1, 3, 5‐year survival rate of PTCLs patients. (B,D)The calibration curves for predicting patient survival at 3 and 5 years in the cohort

In order to develop a convenient clinical tool that could facilitate clinician to predict overall survival (OS) probability of every patient, a nomogram which included a mRNA signature, age and gender was constructed to predict the 1‐, 3‐ and 5‐year OS of PTCLs patients (Figure 7A,C).The calibration curve also illustrates high consistency between predictive survival time and observation survival time for the probabilities of 3‐ and 5‐year OS in the PTCLs cohort. In the GSE58445 dataset, the Harrell's concordance index for OS was 0.722 (Figure 7B).In the GSE90597 dataset, the Harrell's concordance index for OS was 0.684 (Figure 7D), it means that the calibration plots for the 3‐ and 5‐year OS rate were estimated well in entire PTCLs patients.

## 4 DISCUSSION

Peripheral T‐cell lymphoma is an aggressively lymphoproliferative disease that seriously threatens human health, and most patients with PTCLs have a poor prognosis due to the combination of the lack of specific treatment and an aggressive clinical process.^22^ However, molecular risk stratification which based on gene expression profile (GEP) into some type of human cancer has opened an avenue for clinicians to personalized medicine and brought enthusiasm for researchers to applicate to other cancer types.^23^ Until recently, PTCLs were lagged behind in terms of risk classification unfortunately. In the present research, we developed a prognostic signature that based on six genes (DOCK2, GSTM1, H2AFY, KCNAB2, LAPTM5 and SYK) for PTCLs and validated it in internal test datasets and external test datasets. Complementary value of clinical characteristics and molecular were further leveraged and showed that combination of both could accurately predict the overall survival of PTCLs.

There is an increasing application of risk signature used for predicting prognosis of cancer patients due to the carcinogenesis and development of tumours are the interaction of multiple genes.^23^, ^24^ On the other hand, the risk signature based on multigenes usually shows better performance in predicting the prognostic value than an individual gene or clinical characteristic risk classifier.^25^ Therefore, in our study, we built a multi‐mRNA‐based signature with the LASSO Cox regression model to predict overall survival of PTCLs, and the prognostic and predictive accuracy of this signature was assessed in training group and testing patient groups. By utilizing this mRNA signature to the PTCLs patients, significantly statistical difference was depicted in the survival curve between high‐risk group and low‐risk group. Compared with wen yin's report of 5 genes signature predicting the survival of Glioblastoma multiforme and jie zhu's research of 6 genes signature discriminating the high‐risk and low‐risk group of lung cancer,^26^, ^27^ our signature classifier show better performance. Additionally, we also demonstrated the predictive value of the mRNA signature for chemotherapy of vincristine in DLBCL and that may helpful for predicting the sensitivity of PTCLs to vincristine. Moreover, to accurately predict the outcome of each individual PTCLs patient, we combine clinical characteristics and 6‐mRNA signature to construct nomograms, and we had evaluated the calibration of the nomogram according to the calibration curve. In our study, the c‐index for the nomogram in two group patient was 0.722 and 0.684, respectively (3‐, 5‐year OS), significantly higher than previous research that used to predict the prognosis of non‐small‐cell lung cancer patients,^28^ showing that there is distinguished consistency between predicted survival probability and actual survival proportion, and indicating our nomogram that based on the six genes signature is a promising tool for predicting the outcome of PTCLs patients and can be useful for clinicians to implement personalized treatment.

In this context, KCNAB2, H2AFY, DOCK2 and GSTM1 in our signature significant high expression in high‐risk group compare to low‐risk group and related to poor prognosis, and all genes except KCNAB2 in signature have been reported to be involved in cancer. Potassium voltage‐gated channel subfamily A regulatory beta subunit 2 (KCNAB2) is a component of voltage‐dependent potassium channels (KCh) proteins,^29^ and it has been documented that KCh proteins play an important role in controlling tumour cell proliferation in the early stages of G1/S transition and even during.^30^ Among all potassium channel proteins, some have been considered as promising tumour markers, such as KCNK5, KCNQ1OT1, KCNH2 and KCNN4.^31^, ^32^ KCNAB2 in our study significant highly expressed in high‐risk group, indicating it may be involved in the disease progression of peripheral T‐cell lymphoma. KCNAB2 in our mRNA signature almost never be reported in cancer pathogenesis or disease progression may be due to its tissue specificity.^29^ H2AFY is one alternatively exon‐spliced isoform of macroH2A, generally expressed in tissues that with active cell proliferation^33^ and the overexpression of the H2AFY in tumour sample could further increase aggressiveness of tumour cells and gave rise to metastasis by decreased the expression level of SOD3.^34^ It has been reported that H2AFY is significantly augmented in breast cancer cells and hepatocellular carcinoma (HCC) compared with normal control cells,^35^ up‐regulated H2AFY also related to poor prognosis of breast cancer by driving overexpression of HER‐2 and might favour HCC progression through pathway of p38 MAPK.^36^, ^37^ DOCK2 as a guanine nucleotide exchange factor (GEF) belongs to the dedicators of cytokinesis (DOCK) family, which originally identified in hematopoietic cell, and now it is also studied in B‐cell lymphoma and prostate cancer.^38^ DOCK2 has the functions of activating small G proteins such as Rac1/2 and subsequently activates downstream pathways which involved in survival, proliferation and migration of cancer.^39^ It has also been demonstrated that DOCK2 was abnormally elevated expressed in B‐cell lymphoma and the overexpressed DOCK2 correlated with the reduced prognosis of chronic lymphocytic leukaemia.^40^, ^41^ GSTM1 (glutathione S‐transferase M1) is a member of the family of cytosolic GSTs, and the null genotype of GSTM1 has been proven to be associated with risk of colorectal cancer, renal cell carcinoma, oesophageal cancer, nasopharyngeal cancer and bladder cancer.^42^, ^43^, ^44^, ^45^, ^46^ LAPTM5 (lysosomal‐associated protein transmembrane 5) is a membrane protein that can inhibit the expression of T‐cell receptor (TCR) and play a positive role in migration and invasion of ovarian cancer cell but play a negative regulator of T‐cell or B‐cell receptor downstream signalling.^47^, ^48^, ^49^ SYK (spleen tyrosine kinase) is an important component involved in immune receptor signal transduction and is found to be highly expressed in most PTCLs.^50^ Moreover, the inhibitor of SYK was shown to not only inhibit T‐cell lymphoma cell lines proliferation but also induce apoptosis.^51^ In our study, the prognosis of the SYK high‐risk group is better than that of the low‐risk group, which may be attributed to the absent expression of SYK in some lymphoma with worse prognosis.^52^ But it cannot be ruled out that it has a protective effect in some subtype of PTCLs, because it has been reported that SYK has a protective effect in some solid tumour.^53^, ^54^, ^55^

Genomic changes have been shown as the cause of carcinogenic and progression of tumours, but in recent analyses infer that the changes in the tumour microenvironment (TME) are also closely related to cancer prognosis and have influence on the response of immunotherapy.^56^ The high infiltration of B cells in tumours has been demonstrated to be associate with patients prolonged survival^57^ and unique role for B cells in antitumour immunity may be responsible for this phenomenon.^58^ To explore the composition of the immune microenvironment of PTCLs, the scale of value of immune cells in the high‐ and low‐risk groups was calculated and analysed. The proportion of naïve B cells is significant higher in the low‐risk group than in the high‐risk group, which in line with the Javeed Iqbal's research that the signatures of B cell predicted a favourable outcome of PTCLs.^59^ In addition to this, the presence of B cell in tumours could promote immunotherapy response,^60^ and it suggests that low‐risk group PTCLs may be more effective for immunotherapy.

Limitations of the present study should be acknowledged. Firstly, the sample size might not be adequate and may lead to selection bias. Secondly, lack of complete clinical characteristics and absent comprehensive analysis of signature and clinical features. What's more, additional genetic and experimental studies are required to elucidate the mechanism and the function of these genes that are included in signature which in the carcinogenesis and progression of PTCLs. Finally, our results in more larger samples or more external independent datasets need further validation.

## 5 CONCLUSION

In conclusion, this is the first study to investigate the ability of mRNA risk signature as novel prognostic biomarkers for PTCLs. In present research, we identified a six‐mRNA based signature for predicting OS of PTCLs and the mRNA signature has showed power performance to stratify all PTCLs patients into low and high risk group. Moreover, A nomogram which integrated mRNA signature and clinical characteristics potentially offers good value for clinicians implementing personalized therapeutic regimen for patients with PTCLs.

## CONFLICT OF INTEREST

The authors declare that they have no competing interests.

## AUTHOR CONTRIBUTION

**Jiannan Tu:** Conceptualization (equal); Data curation (equal); Formal analysis (lead); Methodology (supporting); Software (equal); Visualization (equal); Writing‐original draft (equal). **Zhixing Kuang:** Data curation (equal); Formal analysis (supporting); Methodology (lead); Project administration (equal); Software (equal); Visualization (equal); Writing‐original draft (equal). **Xiaoliang Xie:** Formal analysis (supporting); Resources (supporting); Visualization (equal); Writing‐original draft (equal). **Shizhen Wu:** Investigation (supporting); Software (supporting); Visualization (supporting); Writing‐original draft (equal). **Ting Wu:** Writing‐original draft (equal). **Shengchi Chen:** Conceptualization (supporting); Funding acquisition (lead); Methodology (supporting); Project administration (lead); Resources (supporting); Software (supporting); Writing‐review & editing (equal).

## Supporting information

Figure S1‐S3

## Data Availability

All data generated or analysed during this study are included in this article.
