# Supplementary material for: Prognostic and predictive value of a mRNA signature in peripheral T‐cell lymphomas: A mRNA expression analysis
Source: J Cell Mol Med. 2020 Dec 1;25(1):84–95. doi: 10.1111/jcmm.15851 (PMC7810961; doi:10.1111/jcmm.15851)
Supplement: Supplementary file 1 — Figure S1‐S3 [file JCMM-25-84-s001.docx]

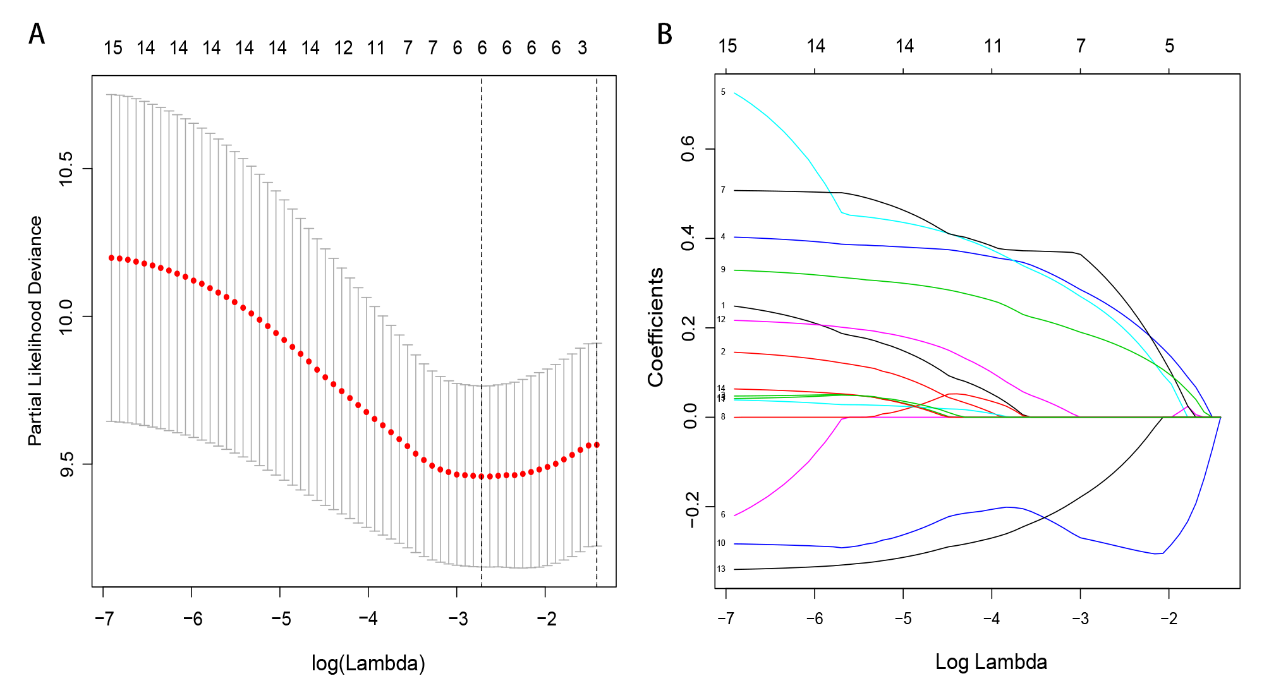


**FIGURE S1** Constructing the six mRNA signature by Lasso cox regression model. (A) Lasso coefficient of the 15 unicox selected genes ;(B) Ten-fold cross-validation for tuning parameter selection in the Lasso module.


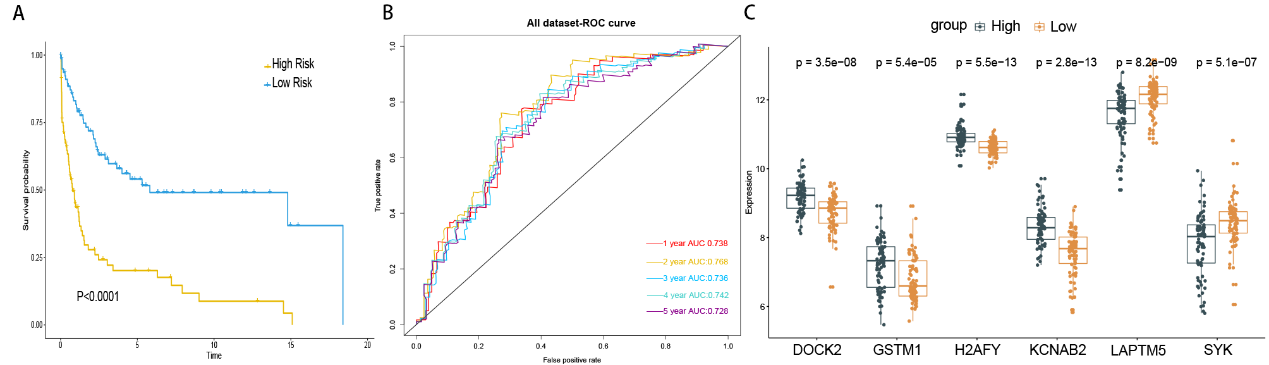


**FIGURE S2** Prognostic and predictive value of the mRNA signature. (A) Kaplan-Meier survival curves for total set of GSE58445 of PTCLs patients. (B) time-dependent ROC curves of 1,2, 3,4, 5years for the six-mRNA signature (C,) Box plot visualization of the expression levels of DOCK2, GSTM1, H2AFY, KCNAB2, LAPTM5 and SYK in different risk group.


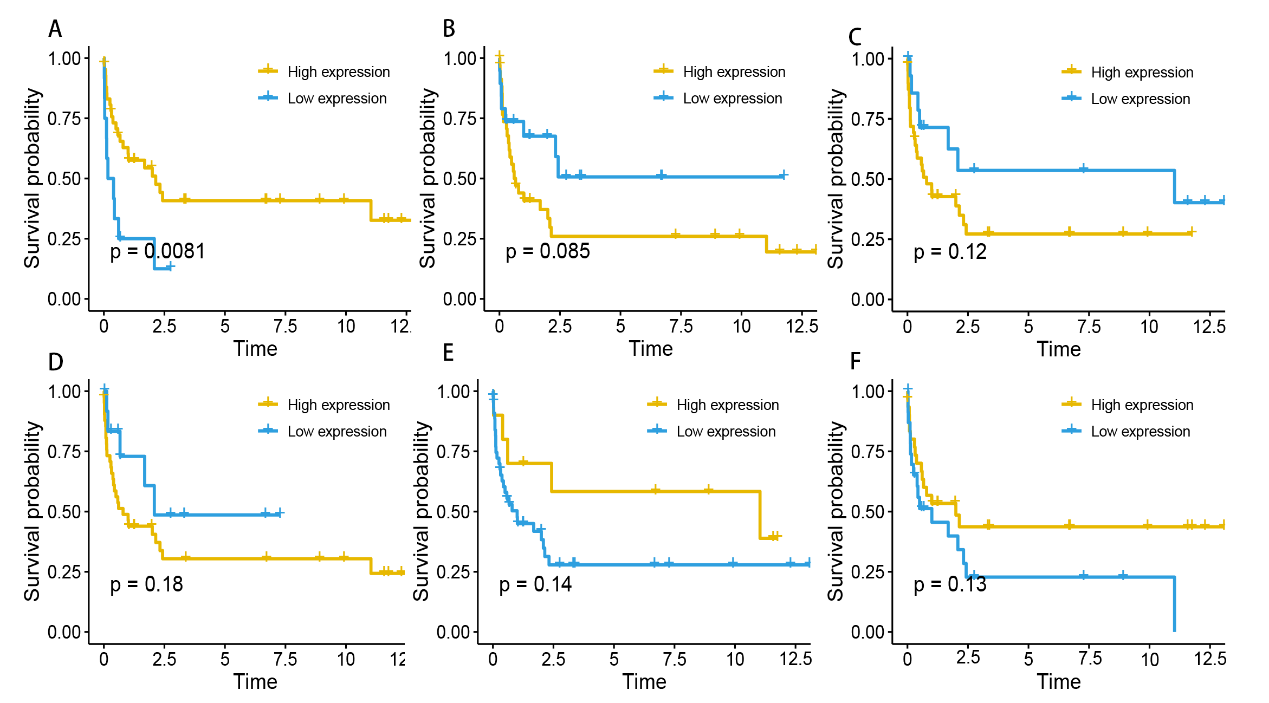


**FIGURE S3** Survival analysis of DOCK2, GSTM1, H2AFY, KCNAB2, LAPTM5 and SYK in GSE90597 PTCLs cohorts (A: DOCK2; B: GSTM1; C:H2AFY; D: KCNAB2; E: LAPTM5; F: SYK).
